# Supplementary material for: Chytrid rhizoid morphogenesis resembles hyphal development in multicellular fungi and is adaptive to resource availability
Source: Proc Biol Sci. 2020 Jun 10;287(1928):20200433. doi: 10.1098/rspb.2020.0433 (PMC7341943; doi:10.1098/rspb.2020.0433)
Supplement: Figures and Tables [file rspb20200433supp13.pdf]

Supplementary Figure 1

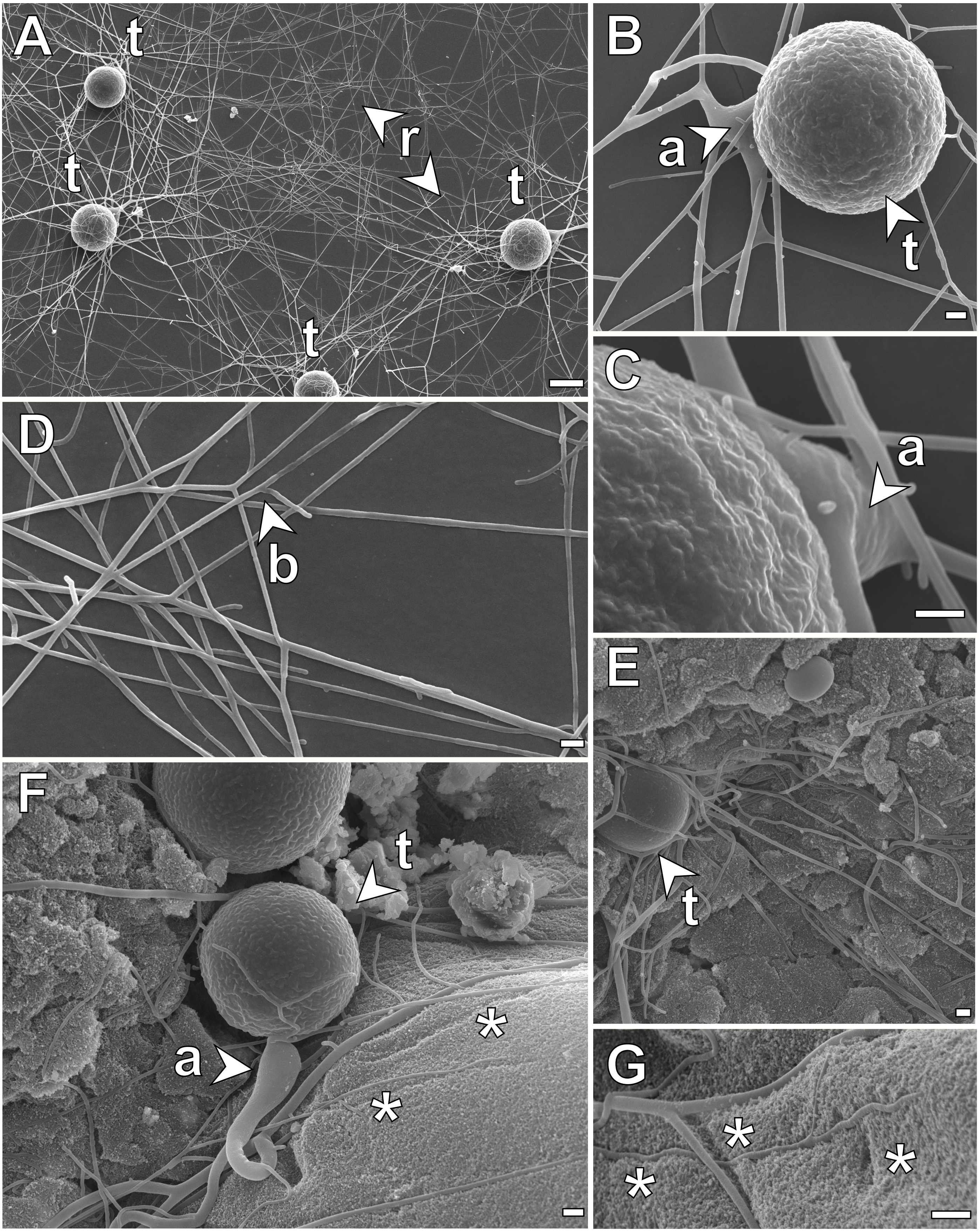

Supplementary Figure 2

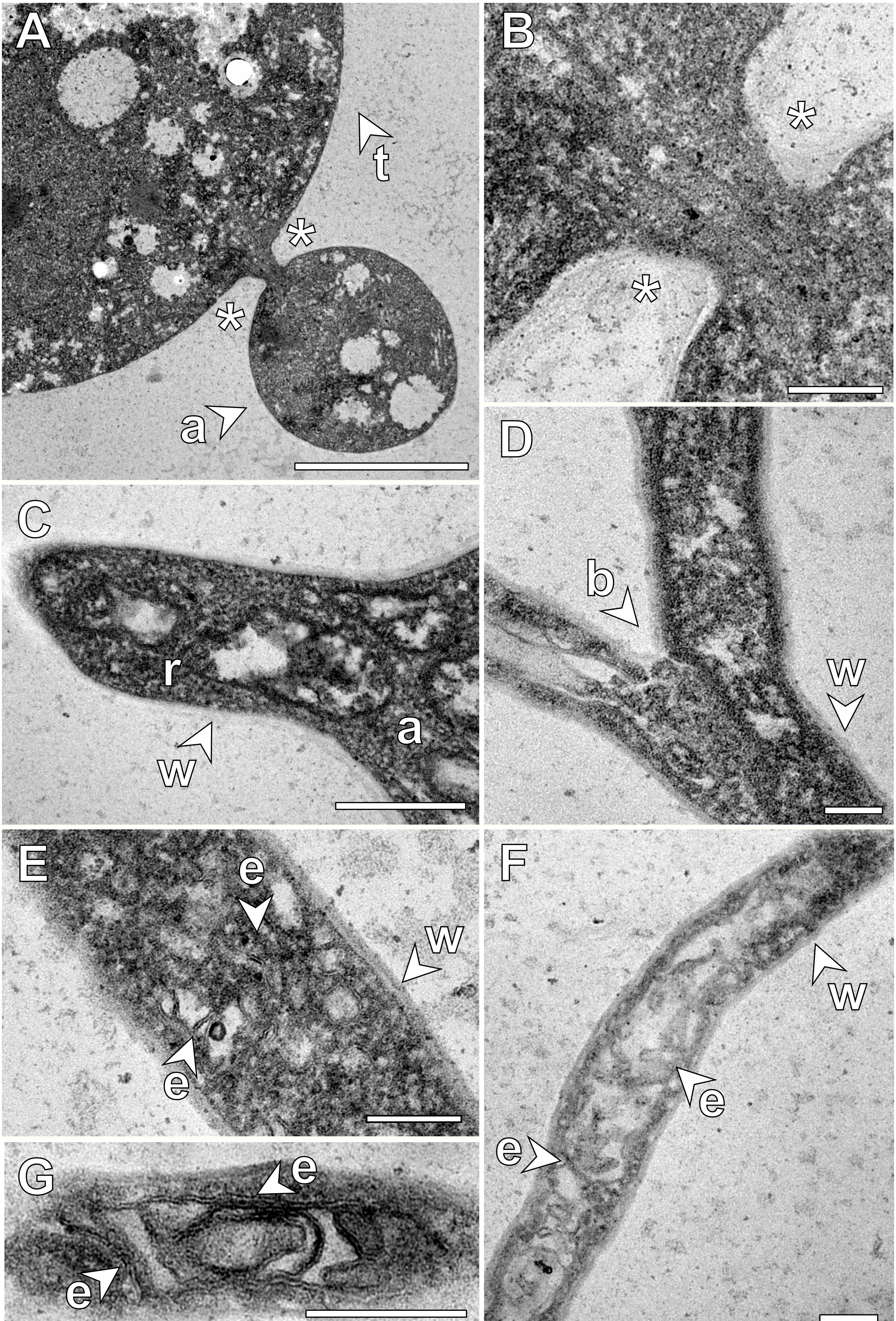

Supplementary Figure 3

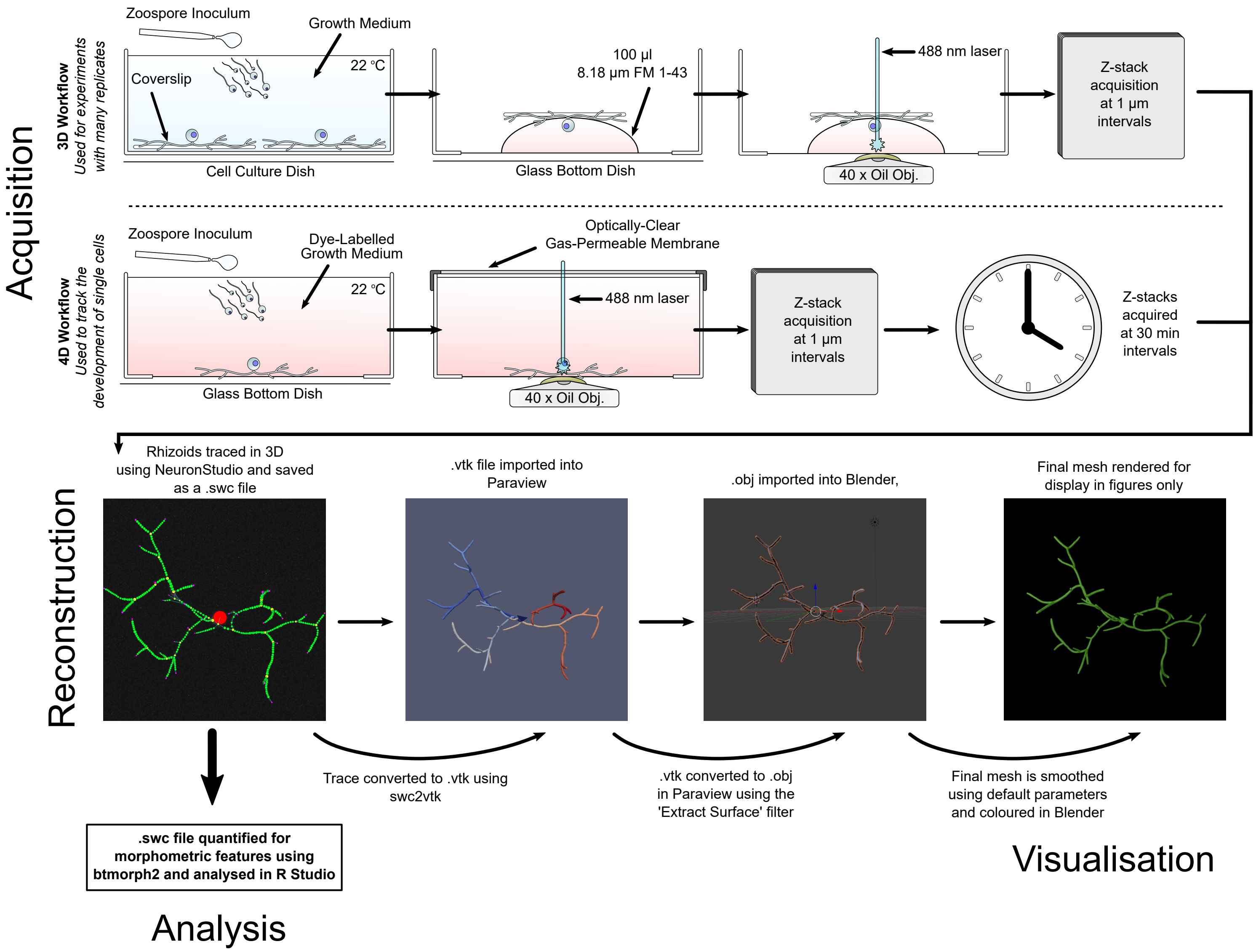

Supplementary Figure 4

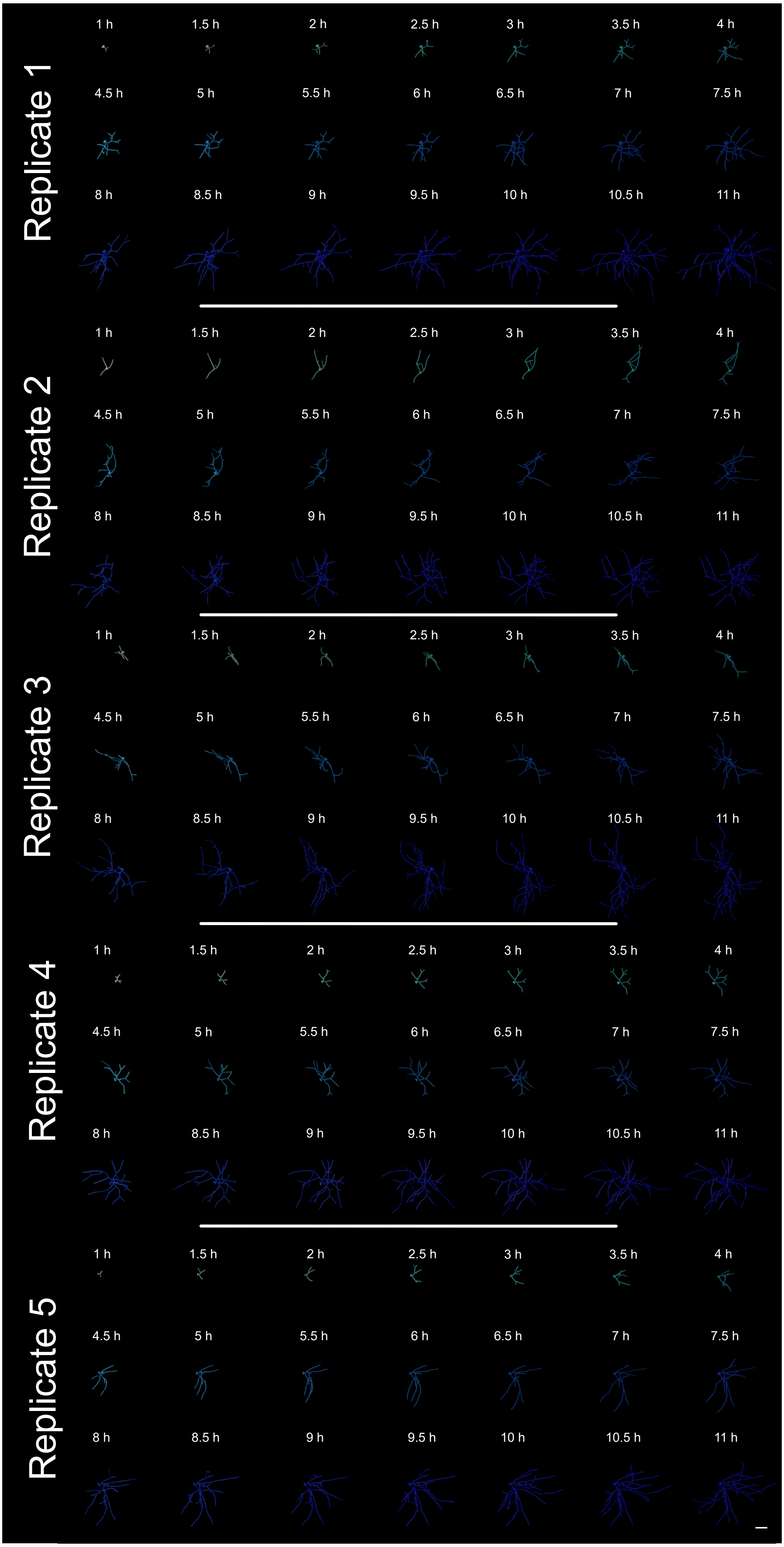

Supplementary Figure 5

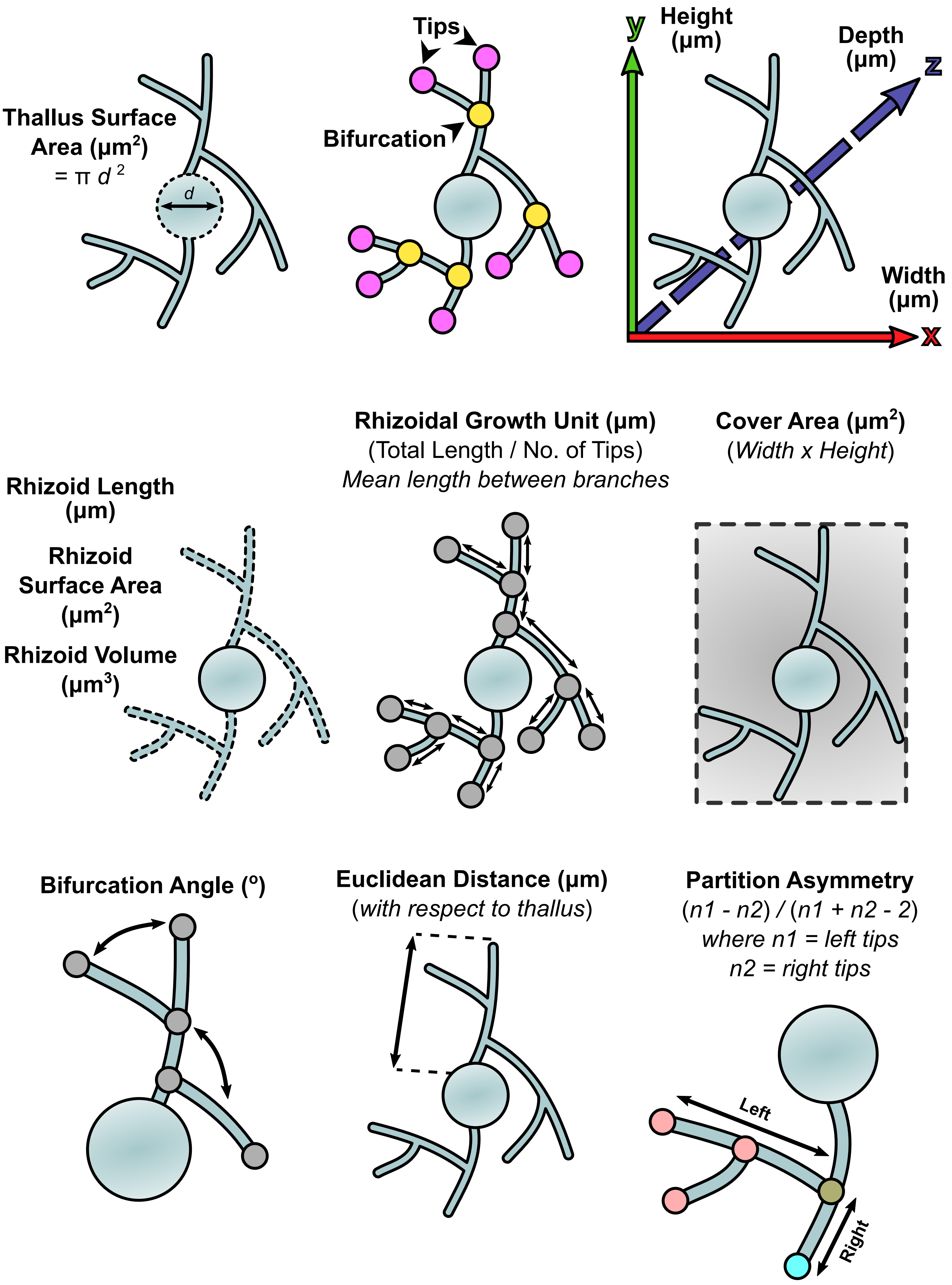

Supplementary Figure 6

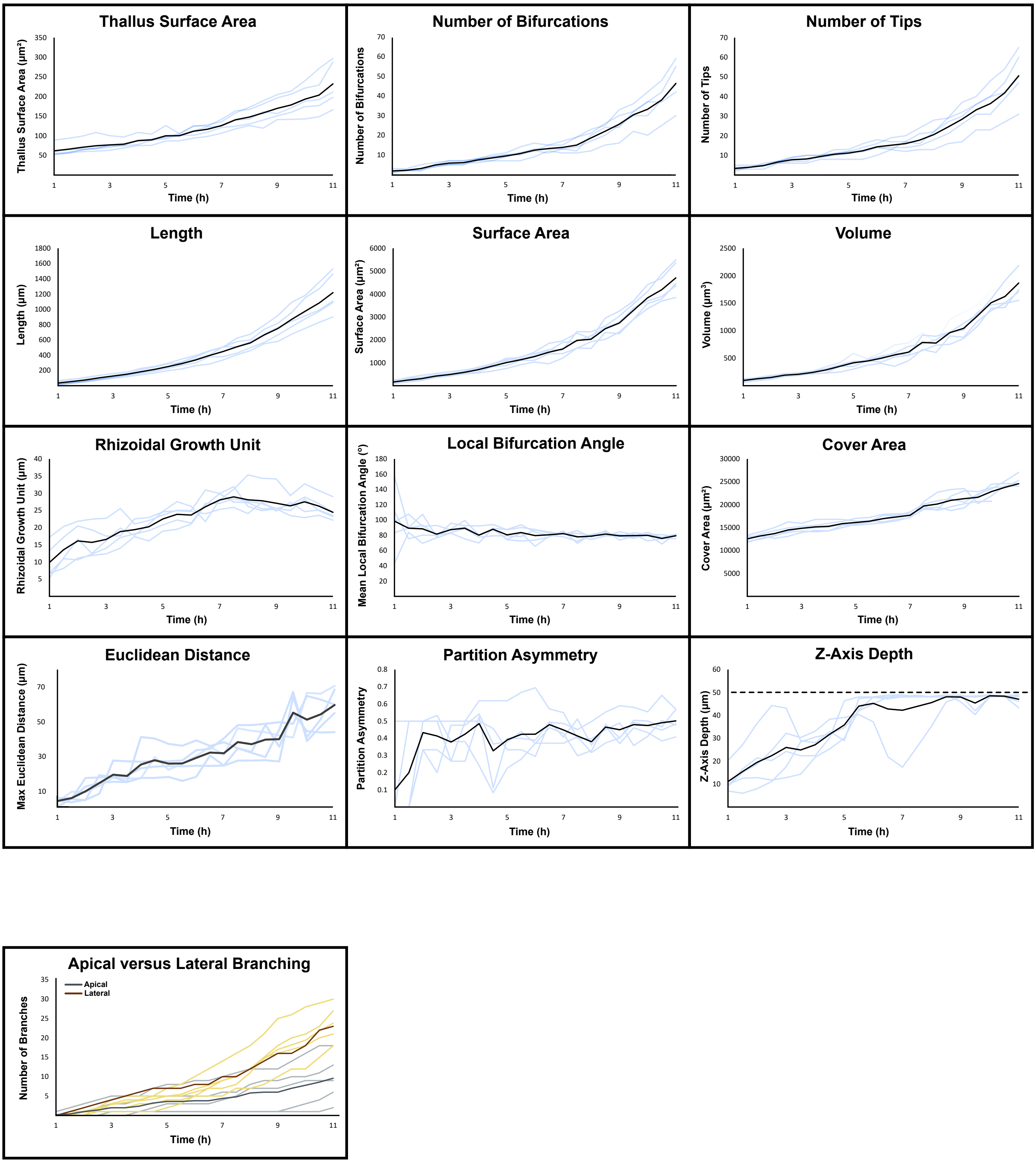

Supplementary Figure 7

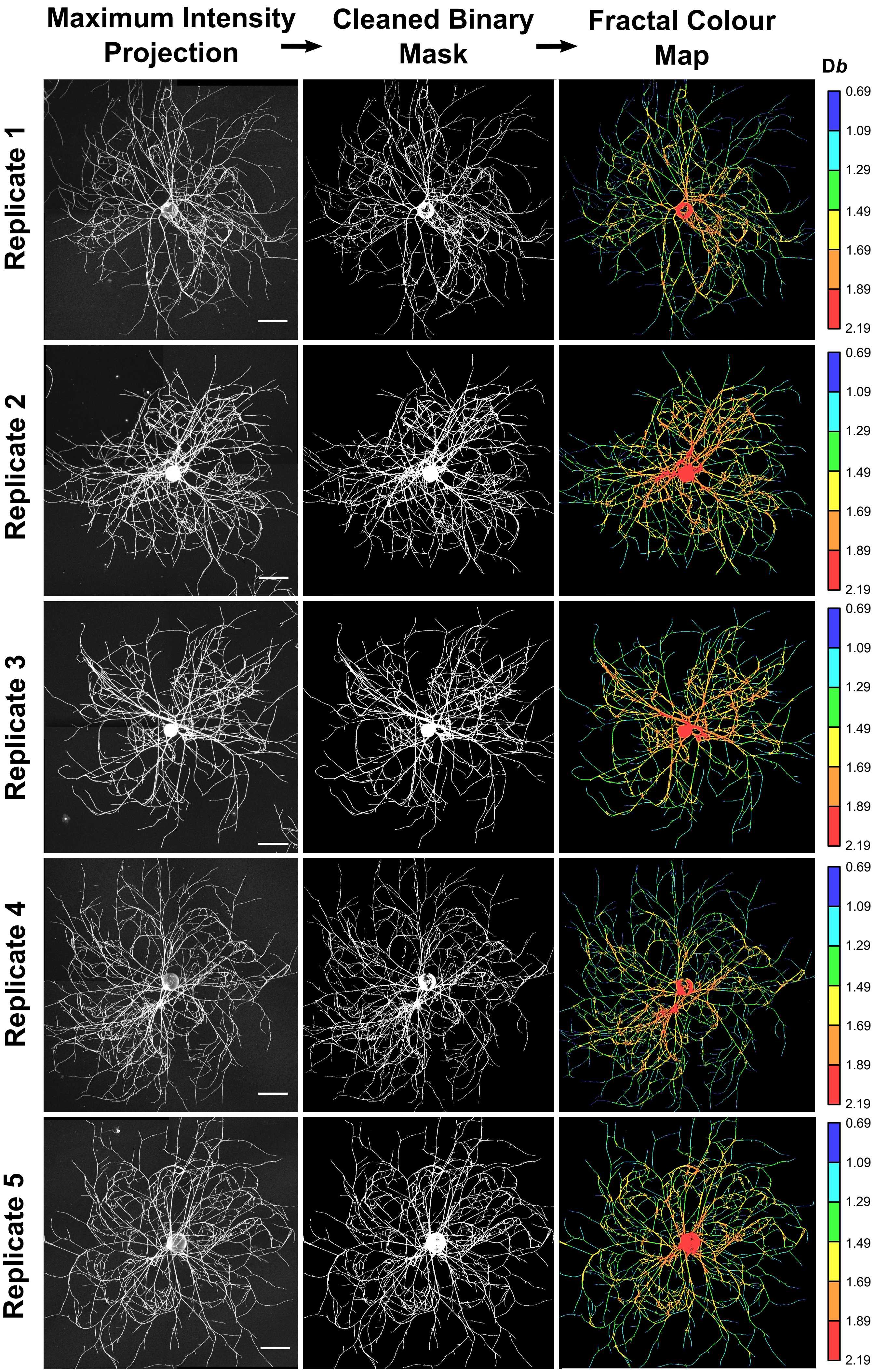

Supplementary Table 1

**4D Development**<br><br>**Morphometric Feature**	**(1 h) (Mean)**	**± Standard Deviation**	**(1.5 h) (Mean)**	**± Standard Deviation**	**(2 h) (Mean)**	**± Standard Deviation**
Thallus Diameter (µm^2)	61.73	15.55	65.73	15.90	70.65	15.91
Number of Bifurcations	1.80	0.84	2.20	0.45	3.00	1.22
Number of Tips	3.40	1.14	4.00	0.71	4.80	1.10
Width (µm)	115.28	2.31	118.40	0.76	120.92	1.52
Height (µm)	108.89	4.02	111.68	5.43	113.31	7.81
Depth (µm)	11.13	5.23	15.48	7.68	19.48	10.83
Total Length (µm)	34.96	23.27	55.49	26.18	76.24	27.57
Surface Area (µm^2)	160.28	67.69	242.43	86.65	309.83	95.17
Volume (µm^3)	95.35	24.87	126.35	24.33	150.28	27.20
Rhizoidal Growth Unit (µm)	9.87	5.15	13.57	5.02	16.16	5.17
Cover Area (µm^2)	12558.93	680.10	13222.81	663.30	13701.19	945.32
Mean Bifurcation Angle (º)	98.56	41.45	89.28	11.85	88.46	14.35
Partition Asymmetry	0.10	0.22	0.20	0.27	0.43	0.09
Max Euclidean Distance (µm)	4.59	2.06	6.24	2.21	10.10	5.36
Max Path Distance (µm)	4.92	2.43	7.21	2.93	11.50	5.88
Number of Apical Branches	0.20	0.45	0.40	0.89	1.00	1.22
Number of Lateral Branches	0.00	0.00	0.40	0.55	0.80	0.84
	**(2.5 h) (Mean)**	**± Standard Deviation**	**(3 h) (Mean)**	**± Standard Deviation**	**(3.5 h) (Mean)**	**± Standard Deviation**
Thallus Diameter (µm^2)	74.64	19.18	80.28	13.97	78.74	10.72

Number of Bifurcations	4.80	0.84	6.00	1.10	6.00	1.00
Number of Tips	6.60	0.55	8.17	1.30	8.20	1.79
Width (µm)	124.56	1.06	126.69	2.62	127.67	2.68
Height (µm)	116.33	9.44	117.53	8.31	118.83	10.80
Depth (µm)	22.43	13.50	24.05	12.05	24.90	6.66
Total Length (µm)	103.01	29.69	129.64	28.92	150.42	26.09
Surface Area (µm^2)	419.67	78.87	498.16	80.13	579.40	69.78
Volume (µm^3)	191.82	25.09	216.88	19.13	239.34	23.15
Rhizoidal Growth Unit (µm)	15.72	4.89	16.14	3.97	18.85	4.29
Cover Area (µm^2)	14489.81	1178.25	14876.19	798.19	15148.16	1056.39
Mean Bifurcation Angle (^{o})	81.51	6.51	85.40	5.20	89.34	8.82
Partition Asymmetry	0.41	0.14	0.39	0.11	0.42	0.10
Max Euclidean Distance (µm)	14.85	4.23	19.28	4.71	18.90	4.97
Max Path Distance (µm)	18.14	5.13	23.20	5.13	23.75	5.18
Number of Apical Branches	1.40	1.67	2.50	2.00	2.00	2.00
Number of Lateral Branches	1.80	0.84	3.00	1.30	3.40	1.82
	**(4 h) (Mean)**	**± Standard Deviation**	**(4.5 h) (Mean)**	**± Standard Deviation**	**(5 h) (Mean)**	**± Standard Deviation**
Thallus Diameter (µm^2)	87.55	13.18	89.84	10.18	99.90	15.33
Number of Bifurcations	7.40	0.89	8.40	1.34	9.40	1.52
Number of Tips	9.40	0.89	10.60	1.82	11.20	1.92
Width (µm)	128.49	4.98	130.81	6.80	133.09	8.08
Height (µm)	119.48	13.29	121.85	11.97	121.57	10.10
Depth (µm)	27.16	5.01	31.81	4.67	35.82	7.13
Total Length (µm)	183.21	34.22	212.60	35.17	250.30	39.92

Surface Area (µm^2)	697.97	112.24	856.05	134.33	1004.34	184.45
Volume (µm^3)	286.28	51.44	351.77	63.07	416.94	108.92
Rhizoidal Growth Unit (µm)	19.40	2.28	20.23	2.71	22.54	2.55
Cover Area (µm^2)	15298.59	1096.41	15874.79	707.32	16119.89	603.53
Mean Bifurcation Angle (^{o})	80.11	7.99	87.90	5.39	80.59	6.54
Partition Asymmetry	0.49	0.14	0.33	0.23	0.39	0.14
Max Euclidean Distance (µm)	25.42	9.73	27.94	8.30	26.01	7.13
Max Path Distance (µm)	30.92	11.74	35.03	10.52	34.40	11.86
Number of Apical Branches	2.40	1.95	3.20	2.68	3.60	2.97
Number of Lateral Branches	3.80	1.92	4.20	2.28	5.00	2.12
	**(5.5 h) (Mean)**	**± Standard Deviation**	**(6 h) (Mean)**	**± Standard Deviation**	**(6.5 h) (Mean)**	**± Standard Deviation**
Thallus Diameter (µm^2)	100.34	7.52	111.63	13.83	116.66	12.51
Number of Bifurcations	10.60	2.51	12.40	2.51	13.20	1.79
Number of Tips	12.40	2.97	14.40	2.97	15.20	2.68
Width (µm)	134.75	8.19	137.86	8.19	141.14	9.67
Height (µm)	122.19	8.54	123.33	8.78	123.15	8.15
Depth (µm)	44.05	3.44	45.24	4.66	42.83	11.70
Total Length (µm)	290.01	48.65	336.86	55.51	394.34	70.39
Surface Area (µm^2)	1131.95	138.18	1268.24	186.91	1460.13	325.84
Volume (µm^3)	448.43	45.57	497.52	79.06	560.30	140.57
Rhizoidal Growth Unit (µm)	23.87	3.07	23.65	2.33	26.07	3.45
Cover Area (µm^2)	16414.85	525.82	16960.99	817.17	17329.43	639.39
Mean Bifurcation Angle (^{o})	83.58	8.62	79.63	9.66	80.75	2.71
Partition Asymmetry	0.42	0.15	0.42	0.16	0.48	0.07

Max Euclidean Distance (µm)	26.12	7.24	29.34	8.08	32.36	4.80
Max Path Distance (µm)	35.29	10.43	39.00	12.77	44.57	8.77
Number of Apical Branches	3.60	2.97	3.80	3.35	3.80	3.35
Number of Lateral Branches	5.40	2.07	6.80	2.17	7.80	2.59
	**(7 h) (Mean)**	**± Standard Deviation**	**(7.5 h) (Mean)**	**± Standard Deviation**	**(8 h) (Mean)**	**± Standard Deviation**
Thallus Diameter (μm^2)	125.80	13.55	140.79	20.30	153.26	21.19
Number of Bifurcations	13.80	2.28	15.00	3.81	19.83	4.28
Number of Tips	16.00	3.08	17.80	4.55	22.00	5.55
Width (µm)	144.14	10.96	149.97	9.68	150.13	11.91
Height (µm)	123.26	6.90	131.91	7.67	136.32	9.30
Depth (µm)	42.26	13.92	43.90	9.92	46.12	5.77
Total Length (µm)	447.68	83.46	507.96	93.67	590.88	85.01
Surface Area (μm^2)	1602.52	321.61	1977.68	343.82	2181.31	275.48
Volume (μm^3)	609.97	132.73	786.87	146.15	847.84	112.19
Rhizoidal Growth Unit (µm)	28.05	1.97	28.96	2.52	27.66	4.29
Cover Area (μm^2)	17710.48	522.73	19733.66	687.82	20439.86	1649.74
Mean Bifurcation Angle (^{o})	82.27	2.76	77.85	6.87	78.96	2.85
Partition Asymmetry	0.45	0.07	0.41	0.08	0.40	0.07
Max Euclidean Distance (µm)	31.95	7.37	38.37	8.62	38.31	8.82
Max Path Distance (µm)	44.88	11.89	53.08	14.99	55.74	13.07
Number of Apical Branches	4.40	3.78	4.80	4.15	6.83	4.76
Number of Lateral Branches	9.00	3.39	10.20	3.49	12.50	3.94

	(8.5 h) (Mean)	± Standard Deviation	(9 h) (Mean)	± Standard Deviation	(9.5 h) (Mean)	± Standard Deviation
Thallus Diameter (μm^2)	158.24	28.84	169.98	28.77	178.45	30.65
Number of Bifurcations	22.00	4.53	25.60	6.50	30.40	5.18
Number of Tips	24.40	5.73	28.40	7.50	33.20	6.53
Width (µm)	153.49	15.50	154.51	15.11	157.71	17.56
Height (µm)	136.95	9.97	138.78	14.09	137.92	9.11
Depth (µm)	48.11	1.32	48.03	1.18	45.40	3.89
Total Length (µm)	664.75	106.31	749.34	131.57	869.09	162.80
Surface Area (μm^2)	2484.50	341.81	2736.00	427.52	3290.47	372.99
Volume (μm^3)	963.75	163.46	1043.59	195.90	1269.05	150.26
Rhizoidal Growth Unit (µm)	27.84	3.78	27.12	3.95	26.35	2.25
Cover Area (μm^2)	20955.39	1838.27	21331.96	1840.64	21626.97	1102.86
Mean Bifurcation Angle (^{o})	81.69	3.23	79.24	4.05	79.52	2.78
Partition Asymmetry	0.47	0.06	0.45	0.09	0.48	0.08
Max Euclidean Distance (µm)	39.80	10.19	40.08	8.86	55.31	10.81
Max Path Distance (µm)	55.92	12.69	57.55	16.95	74.23	12.13
Number of Apical Branches	6.00	4.90	6.00	4.90	7.00	5.48
Number of Lateral Branches	14.60	4.62	17.00	5.39	18.20	5.22
	**(10 h) (Mean)**	**± Standard Deviation**	**(10.5 h) (Mean)**	**± Standard Deviation**	**(11 h) (Mean)**	**± Standard Deviation**
Thallus Diameter (μm^2)	193.39	38.51	203.54	47.69	232.18	57.64
Number of Bifurcations	33.20	8.53	38.00	8.46	46.40	11.41
Number of Tips	36.40	9.61	41.80	10.03	50.60	13.16

Width (µm)	166.25	21.27	168.86	22.61	170.74	25.31
Height (µm)	138.76	12.08	142.45	15.78	145.67	16.12
Depth (µm)	48.55	0.42	48.39	0.30	47.04	2.34
Total Length (µm)	977.64	186.20	1084.53	220.20	1219.15	268.22
Surface Area (µm^2)	3836.65	422.94	4187.25	549.42	4707.64	704.53
Volume (µm^3)	1510.22	145.44	1623.77	209.37	1868.34	280.25
Rhizoidal Growth Unit (µm)	27.51	3.84	26.30	2.78	24.48	2.67
Cover Area (µm^2)	22884.73	1500.84	23811.10	1536.94	24595.64	1879.28
Mean Bifurcation Angle (^{o})	79.91	4.24	75.90	4.53	79.41	2.45
Partition Asymmetry	0.47	0.06	0.49	0.10	0.50	0.07
Max Euclidean Distance (µm)	51.27	13.40	54.37	9.71	59.69	10.78
Max Path Distance (µm)	72.72	13.07	78.64	11.26	86.12	12.58
Number of Apical Branches	7.80	5.97	8.60	6.58	9.60	6.19
Number of Lateral Branches	19.40	5.81	21.80	5.07	23.80	4.76

Supplementary Table 2

1 µM Caspofungin Diacetate Morphometric Feature	Poisoned Cells (Mean)	± Standard Deviation	Control Cells (Mean)	± Standard Deviation	*t*-test *p*-value
Thallus Diameter (µm^2)	156.35	33.59	149.01	12.84	$p > 0.05$
Number of Bifurcations	22.25	5.26	20.75	3.73	$p > 0.05$
Number of Tips	25.63	6.16	23.38	3.29	$p > 0.05$
Width (µm)	171.95	23.36	183.52	20.38	$p > 0.05$
Height (µm)	149.81	16.35	166.01	12.42	$p < 0.05$
Depth (µm)	14.84	5.98	12.85	2.09	$p > 0.05$
Total Length (µm)	558.41	113.78	511.62	117.53	$p > 0.05$
Surface Area (µm^2)	2188.88	922.56	2019.66	312.08	$p > 0.05$
Volume (µm^3)	927.54	561.11	841.85	139.38	$p > 0.05$
Rhizoidal Growth Unit (µm)	22.08	2.13	21.88	3.83	$p > 0.05$
Cover Area (µm^2)	25841.89	4907.30	30447.12	4005.04	$p > 0.05$
Mean Bifurcation Angle (^{o})	83.87	7.23	77.41	4.13	$p > 0.05$
Partition Asymmetry	0.65	0.06	0.61	0.08	$p > 0.05$
Max Euclidean Distance (µm)	58.60	12.95	60.06	13.31	$p > 0.05$
Max Path Distance (µm)	89.46	27.71	69.70	15.94	$p > 0.05$
**10 µM Caspofungin Diacetate Morphometric Feature**	**Poisoned Cells (Mean)**	**± Standard Deviation**	**Control Cells (Mean)**	**± Standard Deviation**	***t*-test *p*-value**
Thallus Diameter (µm^2)	116.66	10.21	146.81	8.06	$p < 0.001$
Number of Bifurcations	16.38	4.21	22.75	4.50	$p < 0.05$
Number of Tips	20.00	4.72	25.50	3.93	$p < 0.05$
Width (µm)	147.69	18.06	178.64	28.42	$p < 0.05$
Height (µm)	117.83	21.04	158.94	27.44	$p < 0.01$
Depth (µm)	9.73	2.93	12.66	2.56	$p > 0.05$
Total Length (µm)	236.11	56.46	507.82	60.22	$p < 0.001$
Surface Area (µm^2)	778.83	168.46	1938.29	176.30	$p < 0.001$
Volume (µm^3)	335.65	62.71	780.04	56.72	$p < 0.001$
Rhizoidal Growth Unit (µm)	11.91	2.20	20.41	4.22	$p < 0.001$
Cover Area (µm^2)	17415.38	4017.82	28280.04	6059.43	$p < 0.01$
Mean Bifurcation Angle (^{o})	82.95	7.60	83.81	5.53	$p > 0.05$
Partition Asymmetry	0.43	0.20	0.69	0.07	$p < 0.01$
Max Euclidean Distance (µm)	27.08	5.83	59.78	8.10	$p < 0.001$
Max Path Distance (µm)	35.56	7.84	67.72	7.82	$p < 0.001$

**50 µM Caspofungin Diacetate Morphometric Feature**	**Poisoned Cells (Mean)**	**± Standard Deviation**	**Control Cells (Mean)**	**± Standard Deviation**	***t*-test *p*-value**
Thallus Diameter (µm^2)	102.66	4.58	138.43	21.87	$p < 0.01$
Number of Bifurcations	12.63	3.38	20.50	8.28	$p < 0.05$
Number of Tips	15.13	3.36	23.38	8.21	$p < 0.05$
Width (µm)	144.14	5.03	207.57	82.07	$p < 0.05$
Height (µm)	116.13	24.38	166.90	81.07	$p < 0.001$
Depth (µm)	7.41	3.17	11.87	2.89	$p < 0.05$
Total Length (µm)	68.02	16.73	409.88	102.22	$p < 0.001$
Surface Area (µm^2)	268.76	58.11	1523.21	576.05	$p < 0.001$
Volume (µm^3)	182.96	20.38	664.00	386.28	$p < 0.001$
Rhizoidal Growth Unit (µm)	4.52	0.64	20.67	13.09	$p < 0.001$
Cover Area (µm^2)	16742.96	3613.72	40161.41	43389.89	$p < 0.01$
Mean Bifurcation Angle (°)	87.60	9.17	86.20	11.15	$p > 0.05$
Partition Asymmetry	0.53	0.16	0.60	0.12	$p > 0.05$
Max Euclidean Distance (µm)	14.68	3.36	43.64	13.05	$p < 0.001$
Max Path Distance (µm)	18.54	4.36	57.03	10.59	$p < 0.001$
**0.1 µM Cytochalasin B Morphometric Feature**	**Poisoned Cells (Mean)**	**± Standard Deviation**	**Control Cells (Mean)**	**± Standard Deviation**	***t*-test *p*-value**
Thallus Diameter (µm^2)	145.42	16.91	152.08	16.20	$p > 0.05$
Number of Bifurcations	21.00	3.64	23.13	4.09	$p > 0.05$
Number of Tips	24.11	3.95	26.63	4.00	$p > 0.05$
Width (µm)	163.22	22.88	165.33	12.90	$p > 0.05$
Height (µm)	166.00	21.01	157.67	41.68	$p > 0.05$
Depth (µm)	7.68	1.63	11.22	2.83	$p < 0.01$
Total Length (µm)	423.42	83.00	449.15	72.78	$p > 0.05$
Surface Area (µm^2)	1631.14	359.82	1653.61	228.71	$p > 0.05$
Volume (µm^3)	684.48	158.14	693.87	128.98	$p > 0.05$
Rhizoidal Growth Unit (µm)	17.87	3.85	17.24	4.13	$p > 0.05$
Cover Area (µm^2)	27253.95	5988.26	26356.21	8234.10	$p > 0.05$
Mean Bifurcation Angle (°)	85.37	8.44	82.42	5.49	$p > 0.05$
Partition Asymmetry	0.65	0.13	0.68	0.09	$p > 0.05$
Max Euclidean Distance (µm)	47.01	11.52	55.56	13.49	$p > 0.05$
Max Path Distance (µm)	59.53	11.47	67.04	16.50	$p > 0.05$
**1 µM Cytochalasin B Morphometric Feature**	**Poisoned Cells (Mean)**	**± Standard Deviation**	**Control Cells (Mean)**	**± Standard Deviation**	***t*-test *p*-value**
Thallus Diameter (µm^2)	151.79	47.97	146.94	25.68	$p > 0.05$

Number of Bifurcations	22.63	11.75	22.13	4.70	$p > 0.05$
Number of Tips	27.38	12.58	25.38	5.07	$p > 0.05$
Width (µm)	155.00	28.00	189.01	16.61	$p < 0.01$
Height (µm)	125.73	33.95	144.44	21.41	$p > 0.05$
Depth (µm)	18.21	5.57	13.09	4.95	$p > 0.05$
Total Length (µm)	355.41	222.00	459.83	72.31	$p > 0.05$
Surface Area (µm^2)	1422.36	1066.42	1994.94	353.44	$p > 0.05$
Volume (µm^3)	663.73	513.32	873.25	178.13	$p > 0.05$
Rhizoidal Growth Unit (µm)	12.41	2.78	18.35	2.05	$p < 0.001$
Cover Area (µm^2)	20192.72	7376.37	27145.56	3479.91	$p < 0.05$
Mean Bifurcation Angle (^{o})	82.90	3.27	81.86	6.59	$p > 0.05$
Partition Asymmetry	0.56	0.06	0.64	0.07	$p < 0.05$
Max Euclidean Distance (µm)	33.95	16.23	48.33	9.11	$p > 0.05$
Max Path Distance (µm)	44.62	17.45	64.48	11.60	$p < 0.05$
**10 µM Cytochalasin B<br><br>Morphometric Feature**	**Poisoned Cells (Mean)**	**± Standard Deviation**	**Control Cells (Mean)**	**± Standard Deviation**	***t*-test *p*-value**
Thallus Diameter (µm^2)	104.17	23.54	124.72	13.06	$p < 0.05$
Number of Bifurcations	10.78	1.79	16.33	3.43	$p < 0.01$
Number of Tips	14.00	2.40	19.22	2.44	$p < 0.001$
Width (µm)	145.38	12.72	179.84	22.04	$p < 0.01$
Height (µm)	103.63	28.11	139.30	16.38	$p < 0.01$
Depth (µm)	8.70	2.56	8.92	2.64	$p > 0.05$
Total Length (µm)	119.58	61.95	319.61	57.12	$p < 0.001$
Surface Area (µm^2)	489.73	237.80	1099.97	250.29	$p < 0.001$
Volume (µm^3)	259.75	97.96	449.04	115.66	$p < 0.01$
Rhizoidal Growth Unit (µm)	8.68	4.52	16.62	2.06	$p < 0.01$
Cover Area (µm^2)	15140.54	4661.50	25114.02	4842.67	$p < 0.001$
Mean Bifurcation Angle (^{o})	90.58	5.79	90.28	7.59	$p > 0.05$
Partition Asymmetry	0.49	0.09	0.62	0.08	$p < 0.01$
Max Euclidean Distance (µm)	18.43	9.74	34.84	10.48	$p < 0.01$
Max Path Distance (µm)	22.91	12.39	52.32	19.61	$p < 0.01$

Supplementary Figure 3

Gene	Function	Query Organism	Uniprot Query	JEL800 Blast Best Hit	Score	Evalue	% ID
FKS1	Hyphal development	*Saccharomyces cerevisiae*	P38631	*No hit*	N/A	N/A	N/A
FKS2	Hyphal development	*Saccharomyces cerevisiae*	P40989	*No hit*	N/A	N/A	N/A
BGS1	Hyphal development	*Schizosaccharomyces pombe*	Q10287	*No hit*	N/A	N/A	N/A
BGS2	Hyphal development	*Schizosaccharomyces pombe*	O13967	*No hit*	N/A	N/A	N/A
BGS3	Hyphal development	*Schizosaccharomyces pombe*	Q9P377	*No hit*	N/A	N/A	N/A
BGS4	Hyphal development	*Schizosaccharomyces pombe*	O74475	*No hit*	N/A	N/A	N/A

Supplementary Table 4

(1 h)<br><br>Morphometric Feature	Carbon Replete (Mean)	± Standard Deviation	Carbon Deplete (Mean)	± Standard Deviation	*t*-test *p*-value
Thallus Diameter (μm^2)	65.33	10.96	51.85	11.85	$p < 0.01$
Number of Bifurcations	4.33	1.58	3.63	2.70	$p > 0.05$
Number of Tips	5.78	1.30	4.75	2.60	$p > 0.05$
Width (µm)	47.59	7.42	42.38	22.60	$p > 0.05$
Height (µm)	49.04	12.71	45.21	20.65	$p > 0.05$
Depth (µm)	7.58	2.60	6.74	1.68	$p > 0.05$
Total Length (µm)	75.54	16.57	74.47	55.59	$p > 0.05$
Surface Area (μm^2)	207.65	36.34	173.69	205.38	$p > 0.05$
Volume (μm^3)	98.70	22.65	70.36	70.72	$p < 0.001$
Rhizoidal Growth Unit (µm)	13.19	1.53	15.82	2.75	$p > 0.05$
Cover Area (μm^2)	2400.60	934.82	1959.75	2892.47	$p > 0.05$
Mean Bifurcation Angle (^{o})	93.94	16.81	92.24	16.11	$p > 0.05$
Partition Asymmetry	0.50	0.13	0.54	0.17	$p > 0.05$
Max Euclidean Distance (µm)	11.26	4.81	13.08	11.37	$p > 0.05$
Max Path Distance (µm)	13.67	5.78	16.05	12.60	$p > 0.05$
**(4 h)<br><br>Morphometric Feature**	**Carbon Replete (Mean)**	**± Standard Deviation**	**Carbon Deplete (Mean)**	**± Standard Deviation**	***t*-test *p*-value**
Thallus Diameter (μm^2)	103.87	16.89	70.28	10.53	$p < 0.001$
Number of Bifurcations	14.38	4.47	10.44	2.88	$p > 0.05$
Number of Tips	16.25	4.33	12.00	2.74	$p < 0.05$
Width (µm)	102.31	17.57	142.60	20.37	$p < 0.001$
Height (µm)	103.86	22.07	152.28	40.59	$p < 0.01$
Depth (µm)	9.35	1.63	7.23	1.68	$p < 0.05$
Total Length (µm)	297.44	91.96	400.84	41.77	$p < 0.05$
Surface Area (μm^2)	1052.25	355.81	1353.25	399.14	$p > 0.05$
Volume (μm^3)	413.31	174.59	440.55	231.56	$p > 0.05$
Rhizoidal Growth Unit (µm)	18.23	2.12	35.06	9.50	$p < 0.001$
Cover Area (μm^2)	10840.47	3982.10	22043.11	7629.16	$p < 0.01$
Mean Bifurcation Angle (^{o})	79.35	8.36	86.91	7.40	$p > 0.05$
Partition Asymmetry	0.60	0.11	0.58	0.09	$p > 0.05$
Max Euclidean Distance (µm)	41.41	10.76	75.16	22.69	$p < 0.01$
Max Path Distance (µm)	47.53	10.83	91.44	23.97	$p < 0.001$

(7 h)<br><br>**Morphometric Feature**	**Carbon Replete (Mean)**	**± Standard Deviation**	**Carbon Deplete (Mean)**	**± Standard Deviation**	***t*-test *p*-value**
Thallus Diameter (µm^2)	179.38	28.07	85.36	85.36	$p < 0.001$
Number of Bifurcations	25.67	6.08	25.56	25.56	$p > 0.05$
Number of Tips	28.11	6.33	26.89	26.89	$p > 0.05$
Width (µm)	173.98	19.14	185.34	185.34	$p > 0.05$
Height (µm)	166.68	26.11	215.32	215.32	$p < 0.05$
Depth (µm)	10.88	3.14	10.85	10.85	$p > 0.05$
Total Length (µm)	635.08	135.09	800.14	800.14	$p < 0.05$
Surface Area (µm^2)	2394.43	484.77	2914.51	2914.51	$p > 0.05$
Volume (µm^3)	982.62	190.12	946.72	946.72	$p > 0.05$
Rhizoidal Growth Unit (µm)	23.00	4.75	31.23	31.23	$p < 0.05$
Cover Area (µm^2)	29227.45	6934.82	39176.52	39176.52	$p < 0.05$
Mean Bifurcation Angle (º)	82.19	6.23	88.03	88.03	$p < 0.05$
Partition Asymmetry	0.63	0.07	0.65	0.65	$p > 0.05$
Max Euclidean Distance (µm)	62.15	7.66	120.92	120.92	$p < 0.01$
Max Path Distance (µm)	75.05	5.18	156.50	156.50	$p < 0.001$
**(24 h)**<br><br>**Morphometric Feature**	**Carbon Replete (Mean)**	**± Standard Deviation**	**Carbon Deplete (Mean)**	**± Standard Deviation**	***t*-test *p*-value**
Thallus Diameter (µm^2)	2038.41	336.41	180.49	24.79	$p < 0.01$
Number of Bifurcations	365.75	80.22	88.00	24.42	$p < 0.01$
Number of Tips	433.25	106.58	90.63	23.74	$p < 0.01$
Width (µm)	402.03	28.77	364.64	48.94	$p < 0.05$
Height (µm)	401.90	15.90	393.74	19.71	$p > 0.05$
Depth (µm)	25.14	5.19	9.69	2.75	$p < 0.01$
Total Length (µm)	9918.81	2094.98	3015.64	815.10	$p < 0.01$
Surface Area (µm^2)	43028.44	9579.73	12093.19	3594.02	$p < 0.01$
Volume (µm^3)	28425.62	4653.46	4245.75	1349.18	$p < 0.01$
Rhizoidal Growth Unit (µm)	23.16	1.92	33.23	2.53	$p < 0.001$
Cover Area (µm^2)	161828.23	16638.78	143817.79	22032.07	$p > 0.05$
Mean Bifurcation Angle (º)	68.06	3.21	81.69	2.63	$p < 0.01$
Partition Asymmetry	0.64	0.00	0.67	0.06	$p > 0.05$
Max Euclidean Distance (µm)	206.91	18.27	181.53	34.94	$p > 0.05$
Max Path Distance (µm)	256.90	24.26	235.21	82.52	$p > 0.05$

Supplementary Table 5

**Particulate Carbon<br><br>Morphometric Feature**	**(1 h) (Mean)**	**± Standard Deviation**	**(4 h) (Mean)**	**± Standard Deviation**	**(7 h) (Mean)**	**± Standard Deviation**	**(24 h) (Mean)**	**± Standard Deviation**
Thallus Diameter (µm^2)	71.38	12.81	83.29	9.78	91.89	16.18	269.71	55.76
Number of Bifurcations	2.22	1.30	8.89	4.70	10.75	4.83	112.75	62.47
Number of Tips	3.22	1.30	10.89	5.49	13.50	6.07	143.50	91.81
Width (µm)	131.18	14.57	152.85	30.43	153.14	17.21	171.85	15.96
Height (µm)	97.68	23.73	105.45	22.29	121.57	33.43	145.96	28.79
Depth (µm)	6.92	2.26	32.81	15.63	42.38	9.70	58.99	10.02
Total Length (µm)	32.95	16.05	191.55	73.31	324.11	108.77	2160.80	722.46
Surface Area (µm^2)	151.11	48.92	715.97	237.15	1164.11	457.21	7260.26	3195.89
Volume (µm^3)	104.61	26.55	291.69	96.99	422.49	177.54	2740.66	1364.26
Rhizoidal Growth Unit (µm)	10.50	4.15	18.97	5.76	25.68	6.64	17.54	5.72
Cover Area (µm^2)	12718.87	2934.84	15959.67	4130.87	18483.84	4996.53	24937.91	4319.79
Mean Bifurcation Angle (^{o})	94.20	18.51	81.76	12.22	92.12	9.37	82.45	1.73
Partition Asymmetry	0.37	0.28	0.40	0.19	0.56	0.07	0.58	0.12
Max Euclidean Distance (µm)	10.43	5.91	52.95	31.98	43.05	17.77	41.48	15.56
Max Path Distance (µm)	12.63	7.17	88.61	61.80	66.92	21.46	63.91	15.28

Supplementary Table 6

**Rhizoid Differentiation**<br><br>**Morphometric Feature**	**Particle Associated (Mean)**	**± Standard Deviation**	**Not Particle Associated (Mean)**	**± Standard Deviation**	***t*-test *p*-value**
Number of Bifurcations	24.63	11.33	28.50	8.65	$p > 0.05$
Number of Tips	30.50	12.75	30.25	8.43	$p > 0.05$
Width (µm)	150.84	22.60	217.94	10.43	$p < 0.001$
Height (µm)	146.25	31.11	201.50	29.49	$p < 0.001$
Depth (µm)	33.32	6.52	9.49	5.17	$p < 0.001$
Total Length (µm)	465.18	167.59	1090.68	310.27	$p < 0.01$
Surface Area (μm^2)	1415.67	623.56	3913.74	1420.74	$p < 0.01$
Volume (μm^3)	406.93	236.86	1291.92	595.80	$p < 0.001$
Rhizoidal Growth Unit (µm)	15.88	4.42	36.16	4.44	$p < 0.001$
Cover Area (μm^2)	22093.22	6323.01	43929.59	6729.19	$p < 0.001$
Mean Bifurcation Angle (^{o})	85.71	9.99	92.76	7.28	$p < 0.001$
Partition Asymmetry	0.54	0.11	0.53	0.15	$p > 0.05$
Max Euclidean Distance (µm)	32.80	11.82	127.76	27.94	$p < 0.001$
Max Path Distance (µm)	85.76	41.84	171.28	57.36	$p < 0.05$

Supplementary Methods

Culture maintenance. *Rhizoclosmatium globosum* JEL800 was maintained on PmTG agar plates (Barr, 1986) at 22 ˚C in the dark. To harvest zoospores for experiments, plates were flooded with 1 ml dH_2O and the suspension passed through a 10 µm cell sieve (pluriSelect). Zoospore density was quantified using a Sedgewick Raft Counter (Pyser SCGI) and a Leica DM1000 (10 x objective) with cells fixed in 2% formaldehyde. Zoospores were diluted to a working density of 6.6 x 10^3 ml^{-1} for all experiments. All experiments detailed below were conducted in Bold's Basal Medium (BBM) supplemented with 1.89 mM ammonium sulfate and 500 $µl.l^{-1}$ F/2 vitamin solution (Guillard & Ryther, 1967).

General cell imaging. Cell plasma membranes were labelled with 8.18 µM FM® 1-43 and imaged using a Zeiss LSM 510 Meta confocal laser scanning microscope (CLSM) (Carl Zeiss) under a 40 x oil-immersion objective lens. For Scanning Electron Microscopy (SEM) of rhizoids growing along a 2D surface, culture dishes were lined with EtOH-sterilised Aclar® disks and filled with 3 ml of BBM with 10 mM N-acetyl-d-glucosamine (NAG), before inoculation with zoospores and incubation for 24 h at 22 °C. For SEM of cells growing on chitin beads, dishes were prepared as described below and were also inoculated and incubated for 24 h. Cells were fixed in 2.5% glutaraldehyde and then rinsed twice in 0.1 M cacodylate buffer (pH 7.2). Fixed samples were dehydrated in a graded alcohol series. Cells were then dried in a Critical Point Drier (K850, Quorum) and attached to SEM sample stubs using carbon infiltrated tabs prior to Cr sputter-coating using a sputter coating unit (Q150T, Quorum). Samples were imaged with a Field Emission Gun Scanning Electron Microscope (JSM-7001F, JEOL) operating at 10 kV. For Transmission Electron

Microscopy (TEM), 24 h cells grown in suspension were fixed as previously described. The samples were secondarily fixed with osmium tetroxide (1%, in buffer pH 7.2, 0.1M) for 1 h, rinsed, and alcohol dehydrated as above. The alcohol was replaced with agar low viscosity resin through a graded resin series. Blocks were sectioned at 50 nm intervals with an ultramicrotome (Ultracut E, Leica) and the sections stained using a saturated solution of uranyl acetate (for 15 min) and Reynold's lead citrate (15 min) before being examined using a transmission electron microscope (JEM-1400, JEOL).

4D rhizoid development. Glass bottom dishes (*n* = 5) containing 3 ml BBM with 10 mM NAG were inoculated with 500 µl zoospore suspension. Zoospores settled for 1h prior to imaging before z-stacks to 50 µm depth were acquired at 30 min time intervals for 10 h at 22 °C. An optically clear film permitting gas exchange covered the dish. Branching was counted manually from maximum intensity projected z-stacks. To quantify rhizoid fractal dimensions, cells were grown on glass bottom dishes for 24 h. Due to the large size of the 24 h cells, z-stacks were stitched together in Fiji (Schindelin *et al.*, 2012) from four individual stacks. Stitched stacks (*n* = 5) were converted to maximum intensity projections, processed into binary masks by default thresholding and denoised. Local Connected Fractal Dimension (LCFD) analysis was conducted using default parameters on binary masks with the Fiji plugin FracLac (Karperien *et al.*, 2013). Rhizoid growth rates (μ) using increase in the total rhizoid length or tip number as growth metrics were quantified as in Trinci (1974), where μ = ([ln (Metric at T2 / Metric at T1)] / [T2-T1]). Metrics for length and number of tips were taken from T = 5 h and T = 10 h, representing the beginning and end of exponential growth respectively. Rhizoid elongation rates (the speed at which

individual rhizoid compartments extend) were quantified by free-hand tracing and measurement of extending rhizoid compartments (ten rhizoids for each biological replicate, $n = 5$) separated by a 30 min interval on maximum intensity projected z-stacks in Fiji. Only rhizoids growing along 2D axes were included to make these results comparable with hyphal studies. To make data comparable for comparison with hyphal fungi, extension rates were also scaled by rhizoid or hyphal diameter from data published by López-Franco et al (1994). Rhizoid diameter for *R. globosum* was 0.23 µm, quantified as the mean measurement of 25 separate rhizoid TEM images.

Rhizoid tracing and reconstruction. Z-stacks of rhizoids were imported into the neuron reconstruction software NeuronStudio (Rodriguez *et al.*, 2006, Rodriguez *et al.*, 2008). Rhizoids were semi-automatically traced with the 'Build Neurite' function using the basal point of the sporangium as the rhizoidal origin. Cells grown for 24 h in BBM 10 mM NAG or on chitin beads were too dense to be manually curated and therefore were automatically traced using dynamic thresholding with a minimum neurite length of 2 µm, although due to their high-density tracings should be considered imperfect. For 4D image stacks, the rhizoid was reconstructed in 3D at each 30 min interval. For particle associated and non-associated rhizoids, traced rhizoid systems from individual cells were manually split into their respective categories.

Rhizoids were exported as SWC file extensions (Stockley *et al.*, 1993) and morphometrically quantified using the btmorph2 library (Torben-Nielsen, 2014) run with Python 3.6.5 implemented in Jupyter Notebook 4.4.0. Reconstructed rhizoids were visualised by converting the SWC files first to VTK files using the swc2vtk

Python script (Daisuke Miyamoto: github.com/ DaisukeMiyamoto /swc2vtk/) and then to OBJ files using the ‘Extract Surface’ filter in ParaView (Ahrens *et al.*, 2005). OBJ files were then imported into Blender (2.79), smoothed using automatic default parameters and rendered for display. OBJ meshes were used for final display only and not analysis. To visualise chitin beads, z-stacks were imported into the Fiji plugin TrakEM2 (Cardona *et al.*, 2012). Chitin beads were manually segmented, and 3D reconstructed by automatically merging traced features along the z-axis. Meshes were then preliminarily smoothed in TrakEM2 and exported as OBJ files into Blender for visualisation.

Chemical characterisation of the rhizoid. To label the cell wall and F-actin throughout the rhizoid system, cells were grown for 24 h in 3 ml BBM with 10 mM NAG on glass bottom dishes. The culture medium was aspirated from the cells, which were then washed three times in 500 µl 1 x PBS (phosphate buffered saline). Cells were subsequently fixed for 1 h in 4% formaldehyde in 1 x PBS and then washed three times in 1 x PBS and once in PEM (100 mM PIPES (piperazine-N,N′-bis(2-ethanesulfonic acid)) buffer at pH 6.9, 1 mM EGTA (ethylene glycol tetraacetic acid), and 0.1 mM $MgSO_4$). Fixed cells were stained with 1:50 rhodamine phalloidin in PEM for 30 min, washed three times in PEM, and finally stained with 5 µg/ml Texas Red-conjugated wheat germ agglutinin (WGA) in PEM for 30 min. Stained cells were further washed three times in PEM and mounted under a glass coverslip with one drop of ProLong^{TM} Gold Antifade Mountant (ThermoFisher). Cells were imaged using the same CLSM as described above with a 63 x oil immersion objective lens. F-Actin was imaged by excitation with a 543 nm HeNe laser and emission at 535-590 nm, and the cell wall by excitation with a 633 nm HeNe laser

and emission at 650-710 nm. No dye controls were run for each excitation/emission channel.

Chemical inhibition of rhizoid growth. Autoclaved glass coverslips (VWR) were placed in a culture dish and submerged in 3 ml BBM with 10 mM NAG. Following 1 h of incubation to allow normal zoospore settlement and germination, 1 ml of growth medium was removed from the dish and 1 ml of poison-containing medium was introduced. Caspofungin diacetate (working concentration 1-50 µM) was used to inhibit cell wall β-glucan synthesis and cytochalasin B (working concentration 0.1-10 µM) was used to inhibit actin filament formation. Cells were further incubated for 6 h, which was found to be sufficient to observe phenotypic variation before being removed from the incubator and held at 4 ˚C prior to imaging. Coverslips were removed from the dishes using EtOH-cleaned forceps and placed cell-side down into a glass bottom dish containing 100 µl of membrane dye.

β-glucan quantification. *R. globosum* was grown to 250 ml in BBM with 10 mM NAG (n = 5) for 7 d before harvesting by centrifugation at 4,700 rpm for 10 min in 50 ml aliquots and washed in 50 ml MilliQ H_2O. The cell pellet from each flask was processed for β-glucans in duplicate using a commercial β-Glucan assay (Yeast & Mushroom) (K-YBGL, Megazyme) following the manufacturer's protocol. A sample of shop-bought baker's yeast was used as a positive control. Glucans were quantified spectrophotometrically using a CLARIOstar® Plus microplate reader (BMG Labtech).

Identification of putative glucan synthases genes. All glycosyl transferase group 2 (GT2) domain-containing proteins within the *R. globosum* genome were identified

using the JGI MycoCosm online portal. GT2 functional domains were identified using DELTA-BLAST (Boratyn *et al.*, 2012) and aligned with MAFFT (Katoh & Standley, 2013). Maximum Likelihood phylogenies were calculated with RAxML (Stamatakis, 2014) using the BLOSUM62 matrix and 100 bootstrap replicates and viewed in FigTree (Andrew Rambaut: github.com/rambaut/figtree/). Overall protein architecture was displayed using genoplotR (Guy *et al.*, 2010).

Carbon starvation and growth on chitin beads. To quantify differential rhizoidal growth under carbon replete and carbon deplete conditions, coverslips were placed in a culture dish and submerged in 3 ml growth medium (either carbon-free BBM or BBM with 10 mM NAG). Dishes were then inoculated with zoospores and incubated for either 1, 4, 7 or 24 h, with the 24 h cell z-stacks stitched as described in the fractal analysis. For both sets of experiments, cells were imaged as per the chemical inhibition experiments above.

Chitin beads (New England Biolabs) were washed three times in carbon-free BBM using a magnetic Eppendorf rack and suspended in carbon-free BBM at a working concentration of 1:1,000 stock concentration. Glass bottom dishes containing 3 ml of the diluted beads were inoculated with zoospores and incubated for either 1, 4, 7 or 24 h prior to imaging. For imaging, the culture medium was aspirated off and beads were submerged in 100 µl FM® 1-43. To understand rhizoid development in a starved cell that had encountered a chitin bead, we imaged cells that contacted a chitin bead following development along the glass bottom of the dish.

Statistical Analysis*.* The comparison between apical and lateral branching was conducted using a Wilcoxon Rank Sum test. Univariate differences in rhizoid morphometrics between experimental treatments were evaluated using Welch's t-tests unless stated otherwise. Shapiro-Wilk and Levene's tests were used to assess normality and homogeneity of variance respectively. If these assumptions could not be met, then Wilcoxon Rank Sum was used as a nonparametric alternative. Univariate morphometric differences between particle-associated and non-associated rhizoids were evaluated using a paired t-test. All data were analysed in RStudio v1.1.456. (R-Studio Team, 2015).

References

Ahrens JB, Geveci B & Law C (2005) ParaView: An End-User Tool for Large-Data Visualization, in *Visualization Handbook*, Hansen CD & Johnson CR, Editors. Butterworth-Heinemann: Burlington: 717-731.

Barr DJS (1986) *Allochytridium expandens* Rediscovered: Morphology, Physiology and Zoospore Ultrastructure*. Mycologia* **78**(3): 439-448.

Boratyn GM, Schäffer AA, Agarwala R *et al.* (2012) Domain enhanced lookup time accelerated BLAST*. Biology Direct* **7**(1): 12.

Cardona A, Saalfeld S, Schindelin J, *et al.* (2012) TrakEM2 Software for Neural Circuit Reconstruction*. PLoS one* **7** (6): e38011.

Guillard RRL & Ryther JH (1967) Studies of marine planktonic diatoms. I. C*yclotella nana* Hustedt and *Detonula confervaceae* (Cleve) Gran. *Canadian Journal of Microbiology* **8**: 229-239.

Guy L, Kultima JR & Andersson SGE (2010) genoPlotR: comparative gene and genome visualization in R. *Bioinformatics* **26**(18): 2334-2335.

Karperien AH, Ahammer H, & Jelinek H (2013) Quantitating the subtleties of microglial morphology with fractal analysis. *Frontiers in Cellular Neuroscience*, **7**(3).

Katoh K & Standley DM (2012) MAFFT Multiple Sequence Alignment Software Version 7: Improvements in Performance and Usability. *Molecular Biology and Evolution* **30**(4): 772-780.

López-Franco R, Bartnicki-Garcia S, Bracker CE (1994) Pulsed growth of fungal hyphal tips. *Proceedings of the National Academy of Sciences* **91**: 12228-12232.

Rodriguez A, Ehlenberger DB, Hof PR, *et al.* (2006) Rayburst sampling, an algorithm for automated three-dimensional shape analysis from laser scanning microscopy images. *Nature Protocols* **1**(4): 2152-2161.

Rodriguez A, Ehlenberger DB, Dickstein DL *et al.* (2008) Automated Three-Dimensional Detection and Shape Classification of Dendritic Spines from Fluorescence Microscopy Images. *PLoS one* **3**(4): e1997.

R-Studio Team (2015) RStudio: Integrated Development for R. Boston, MA: RStudio, Inc.

Schindelin J, Arganda-Carreras I, Frise E, *et al.* (2012) Fiji: an open-source platform for biological-image analysis. *Nature Methods* **9**: 676-682

Stamatakis A (2014) RAxML version 8: a tool for phylogenetic analysis and post-analysis of large phylogenies. *Bioinformatics* **30**(9): 1312-1313.

Stockley EW, Cole HM, Brown AD, *et al.* (1993) A system for quantitative morphological measurement and electrotonic modelling of neurons: three-dimensional reconstruction. *Journal of Neuroscience Methods* **47**(1): 39-51.

Torben-Nielsen B (2014) An Efficient and Extendable Python Library to Analyze Neuronal Morphologies. *Neuroinformatics* **12**(4): 619-622.

Trinci APJ (1974) A Study of the Kinetics of Hyphal Extension and Branch Initiation of Fungal Mycelia. Microbiology **81**: 225-236.
